# Comparative pharmacokinetics of oxyresveratrol alone and in combination with piperine as a bioenhancer in rats

**DOI:** 10.1186/s12906-019-2653-y

**Published:** 2019-09-02

**Authors:** Dhirarin Junsaeng, Tosapol Anukunwithaya, Phanit Songvut, Boonchoo Sritularak, Kittisak Likhitwitayawuid, Phisit Khemawoot

**Affiliations:** 1 Department of Pharmacology and Physiology, Faculty of Pharmaceutical Sciences, Chulalongkorn University, Bangkok, Thailand; 2 Department of Pharmacognosy and Pharmaceutical Botany, Faculty of Pharmaceutical Sciences, Chulalongkorn University, Bangkok, Thailand; 3 Department of Biochemistry and Microbiology, Faculty of Pharmaceutical Sciences, Chulalongkorn University, Bangkok, 10330 Thailand; 4 Preclinical Pharmacokinetics and Interspecies Scaling for Drug Development Research Unit, Chulalongkorn University, Bangkok, Thailand

**Keywords:** *Artocarpus lacucha*, Moraceae, Oxyresveratrol, Piperine, Bioenhancer, Pharmacokinetics

## Abstract

**Background:**

Oxyresveratrol is a major bioactive component derived from the heartwood of *Artocarpus lacucha*. This compound exerts several biological activities, including neuroprotective effects in vitro and in vivo. However, there is limited pharmacokinetic information on this compound, especially its distribution in neuronal tissue and its route of excretion. The aim of this study was to investigate the pharmacokinetic profiles of oxyresveratrol alone and in combination with piperine as a bioenhancer in rats.

**Methods:**

Male Wistar rats were administered with oxyresveratrol 10 mg/kg, oxyresveratrol 10 mg/kg plus piperine 1 mg/kg via intravenous or oxyresveratrol 100 mg/kg, oxyresveratrol 100 mg/kg plus piperine 10 mg/kg via oral gavage. Plasma, internal organs, urine, and feces were collected. Determination of the oxyresveratrol concentration in biological samples was performed by liquid chromatography tandem mass spectrometry.

**Results:**

The combination with piperine had shown a significantly higher maximum concentration in plasma approximately 1500 μg/L within 1–2 h after oral dosing, and could increase oral bioavailability of oxyresveratrol approximately 2–fold. Oxyresveratrol could widely distributed most of the internal organs with a tissue to plasma ratio of 10–100 fold within 5 min after dosing. Urinary excretion of oxyresveratrol glucuronide was the major route of excretion after administration of oxyresveratrol alone and in combination with piperine.

**Conclusion:**

The addition of piperine could enhance some of the pharmacokinetic properties of oxyresveratrol via both intravenous and oral administration. This pharmacokinetic information will be useful for appropriate strategies to develop oxyresveratrol as a phytopharmaceutical product.

**Electronic supplementary material:**

The online version of this article (10.1186/s12906-019-2653-y) contains supplementary material, which is available to authorized users.

## Background

**Fig. 1:**
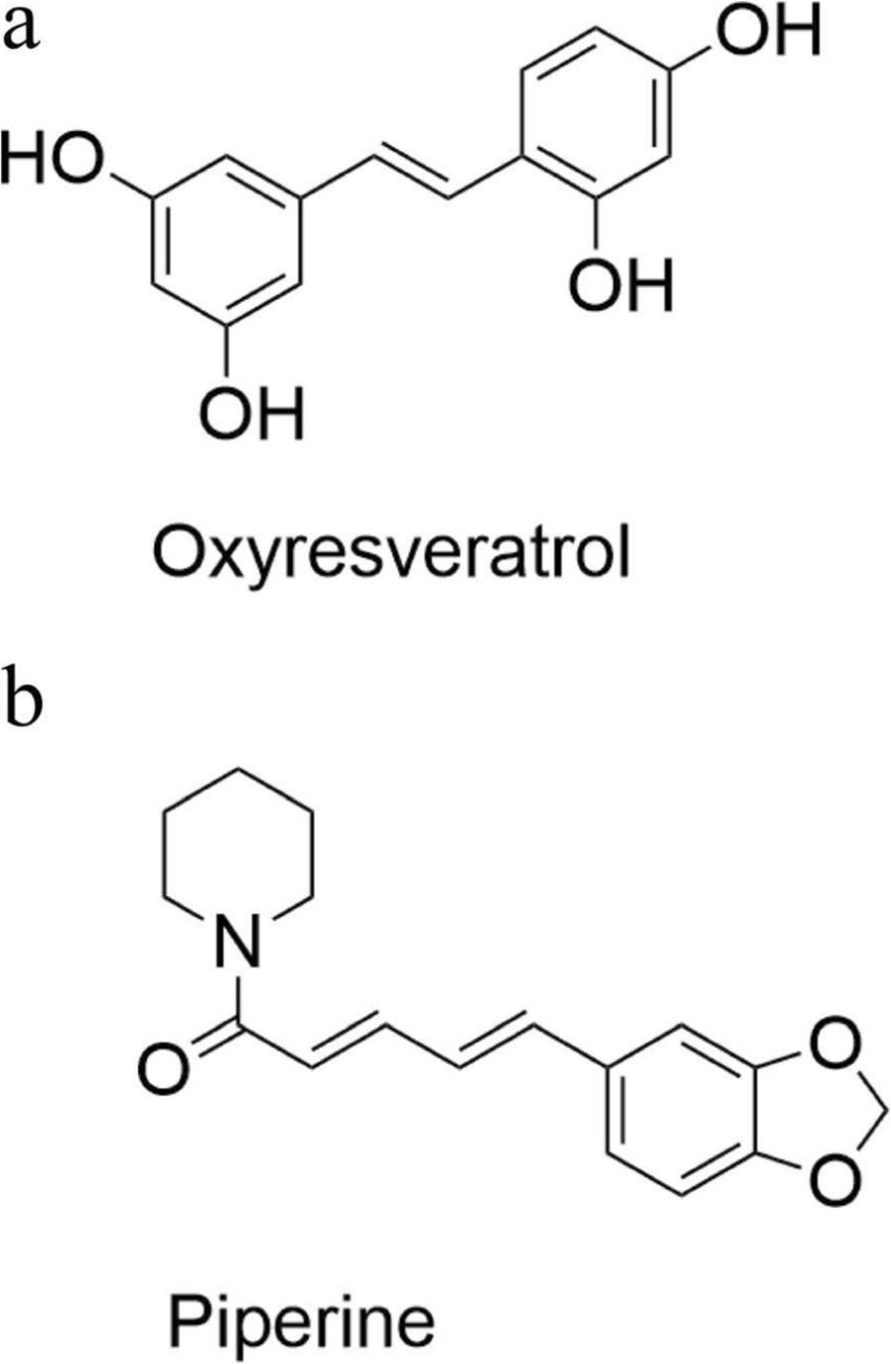
Chemical structures of Oxyresveratrol (**a**); Piperine (**b**)

*Artocarpus lacucha* Buch.–Ham. has several pharmacological activities, and its major bioactive component is oxyresveratrol (2,4,3′,5′–tetrahydroxystilbene, Fig. 1). This compound can inhibit tyrosinase, the major enzyme involved in melanin production in humans, which can have a skin whitening effect [1–3]. Moreover, oxyresveratrol has many other pharmacological activities such as antioxidant [4], anti–inflammatory [5, 6] and antiviral [7, 8] effects, as well as a demonstrated neuroprotective effect in vitro [9]. Oxyresveratrol does not show mutagenic activity within the concentration range of 5–100 μg/mL [10]. Therefore, this compound might have potential for further *vivo* study and development as a phytopharmaceutical product for clinical use. Chen et al. [11] performed a pharmacokinetic study of oxyresveratrol in rats by oral dosing at 100–400 mg/kg. Interestingly, they reported absolute oral bioavailability of approximately 10–15%, with a rapid T_max_ occurring approximately 15 min after dosing. Breuer et al. [12] investigated the tissue distribution of oxyresveratrol at 40 mg/kg after intraperitoneal administration in rats. They found that this compound mainly resided in plasma, and only a minimal concentration reached the brain. Mei et al. [13, 14] reported that oxyresveratrol was biotransformed by phase ΙΙ conjugation, especially glucuronidation, with the majority of metabolites excreted in urine within 12 h of dosing.

Recently, there have been attempts to improve the oral bioavailability and tissue distribution of oxyresveratrol by physical modifications or by combination with bioenhancers. Regarding physical modifications, Sangsen et al. developed a self–microemulsifying drug delivery system (SMEDDS) of oxyresveratrol that could improve the oral bioavailability by 7.9–fold and prevented amyloid–β peptide–induced neurodegeneration in mice [15]. Interestingly, Johnson et al. [16] reported that a combination of resveratrol and piperine could enhance the pharmacokinetic profile of resveratrol, including increases in AUC and C_max_ by up to 229 and 1544%, respectively. Resveratrol has a similar chemical structure to oxyresveratrol [17]; therefore, it is possible that piperine could also enhance the pharmacokinetic profile of oxyresveratrol. In addition, Suresh and Srinivasan [18] reported that piperine reduces the hepatic microsomal activity of UGT in vitro. Furthermore, Atal et al. [19] found that piperine at doses of 10 and 25 mg/kg could decrease in vivo UGT activity by 36 and 55%, respectively. Piperine can also inhibit P–glycoprotein, a major efflux transporter found in the hepatobiliary system and in enterocytes [20].

This study aimed to investigate the pharmacokinetic profiles of oxyresveratrol alone and in combination with piperine in male Wistar rats. Doses of oxyresveratrol and piperine were based on their pharmacodynamic activity and bioenhancer activity, respectively [16]. Oxyresveratrol (100 mg/kg p.o. or 10 mg/kg i.v.) was administered alone or combined with piperine (10 mg/kg p.o. or 1 mg/kg i.v., respectively). The tissue kinetics of oxyresveratrol in pharmacologically relevant organs was also measured to determine the tissue to plasma ratio of the test compounds. Glucuronide metabolites were analyzed in plasma and excreta in order to determine the major routes of excretion after dosing. The pharmacokinetic information obtained from this study will be useful for appropriate strategies to develop oxyresveratrol as a phytopharmaceutical product.

## Materials and methods

### Chemicals

Oxyresveratrol (purity > 98%) and piperine (purity > 98%) for pharmacokinetic experiments were provided by Professor Kittisak Likhitwitayawuid from the Department of Pharmacognosy and Pharmaceutical Botany, Faculty of Pharmaceut ical Sciences, Chulalongkorn University. Analytical grade oxyresveratrol (purity ≥97%) and piperine (purity ≥97%) were purchased from Sigma–Aldrich Corp. Glycyrrhetinic acid (purity > 98%) as the IS for LC–MS/MS analysis was purchased from Wako Pure Chemical Industries, Ltd. DMSO, used as a co–solvent, was purchased from Sigma–Aldrich Corp. NSS, used as vehicle for test compound preparation, was purchased from General Hospital Products. β–Glucuronidase from *Escherichia coli* was purchased from Sigma–Aldrich Corp.

### Animals

Male Wistar rats aged 8–12 weeks old were purchased from Nomura Siam International. The rats were acclimatized at 24 ± 2 °C and 40–60% humidity, under a 12–h dark–light cycle, for at least 1 month prior to the experiments. All animal had ad libitum access to food and water*.* The sample size for pharmacokinetic study was calculated based on Charan and Kantharia [21]. The animal protocol was approved by the Institutional Animal Care and Use Committee of the Faculty of Pharmaceutical Sciences, Chulalongkorn University (approval no. 17–33–002, approved March 8, 2017).

### Pharmacokinetic experiments

Rats weighing more than 400 g were enrolled in the pharmacokinetic experiments. All rats were placed into metabolic cages and fasted overnight before the pharmacokinetic experiments. The rats were divided randomly into four groups (*n* = 6 in each): oxyresveratrol 100 mg/kg p.o., oxyresveratrol 100 mg/kg plus piperine 10 mg/kg p.o., oxyresveratrol 10 mg/kg i.v., and oxyresveratrol 10 mg/kg plus piperine 1 mg/kg i.v. All test formulations were freshly prepared by dissolving the compounds in 40% DMSO/NSS to obtain clear solution. The preparations were administered by oral gavage or intravenous administration via the lateral tail vein. All rats were anaesthetized with 5% isoflurane by chamber induction method to prevent pain and injury during drug administration and blood collection. The blood collection was conducted at 0, 0.083 (5 min), 0.25 (15 min), 0.5 (30 min), 1, 2, 4, 8, 16 and 24 h in unconscious rats. For tissue collection, euthanization with overdose isoflurane > 10% by chamber induction method was conducted at 0.083 (5 min), 1, 2 and 4 h after oral gavage or intravenous administration, and death was confirmed by exsanguination. Excreta from each rat were separately collected at 0–24 h and 24–48 h after dosing. Plasma samples were collected at baseline (0 h) and 24 h from a subset of rats for determination of AST, ALT and creatinine concentrations, performed by Professional Laboratory Management Crop Co., Ltd. Determination of the creatinine level was performed by the chemiluminescence method, while AST and ALT determination was performed with a kinetic method and measured using an automated analyzer (Cobas 6000; Hoffmann–La Roche, Ltd.).

### Sample preparation

The collected blood samples were centrifuged at 5000×*g* for 10 min to collect plasma. Tissue samples were washed with cold NSS and connective tissue was removed. Rat urine was collected from the metabolic cage. Urine was centrifuged at 5000×*g* for 10 min, then 100 μL of urine supernatant was collected and diluted 10–fold with methanol. Rat feces was collected and mixed with methanol up to 10 mL. All biological samples were stored at − 20 °C until analysis. The protein precipitation method with methanol was used to prepare samples for LC–MS/MS analysis. For sample preparation, 50 μL of plasma or urine was mixed with 200 μL of methanol containing 10 ng of IS. The mixture was centrifuged at 10,000×*g* for 10 min, then 150 μL of supernatant was collected and injected into the LC–MS/MS system. Fecal or tissue samples (50 mg) were mixed with 200 μL of methanol containing 10 ng of IS. The mixture was homogenized in an ice bath, then centrifuged at 10,000×*g* for 10 min. In the case of analytes which exceeded the linear calibration curve, the sample was diluted with blank matrices before protein precipitation.

Identification of oxyresveratrol glucuronide was conducted by an indirect method. Briefly, all biological samples were incubated with 2000 units of glucuronidase in phosphate buffer (pH 6.8) at 37 °C for 15 min. The reaction was stopped by adding 1000 μL of methanol containing 50 ng of IS. The mixture was mixed and centrifuged at 10,000×*g* for 10 min, and 150 μL of supernatant was collected for LC–MS/MS analysis.

### Liquid chromatography tandem mass spectrometry (LC–MS/MS) analysis

Quantification of oxyresveratrol and piperine concentrations were carried out following the methods described by Huang et al. [22] and Basu et al. [23], respectively. Briefly, LC–MS/MS was conducted using a Nexera Ultra High–Performance Liquid Chromatography and 8060 triple quadrupole mass spectrometer (Shimadzu Co., Ltd.). The stationary phase was a Synergi Fusion–RP C18 column (Phenomenex Inc.) with an oven temperature of 40 °C. The gradient for the mobile phase was 0.2% formic acid in water and 100% methanol. The gradient started with 50% methanol from 0 to 0.50 min, increased to 90% methanol from 0.50 to 1.50 min, maintained at 90% methanol from 1.50 to 3.00 min, decreased to 50% methanol from 3.00 to 4.00 min, then maintained at 50% methanol from 4.00 to 5.00 min. The retention times for oxyresveratrol, piperine and glycyrrhetinic acid were 0.51, 1.81 and 2.30 min, respectively. Detection of oxyresveratrol and the IS was conducted in negative mode with a mass–to–charge ratio of 245/107 and 469/409 m/z, respectively. Meanwhile, detection of piperine was conducted in positive mode with a mass–to–charge ratio of 286/201 m/z. All chromatograms were free from interference by endogenous substance, as shown in the Additional file 1. The lower limits for quantification of oxyresveratrol and piperine were 6.10 and 0.61 μg/L, respectively. The calibration curve for oxyresveratrol showed a good linearity range from 6.10–12,500 μg/L, and piperine also had a good linearity range from 0.61–1250 μg/L (R^2^ > 0.99). The accuracy and precision of oxyresveratrol and piperine were within ±10%, and the percentage recovery of the extraction method for oxyresveratrol and piperine was higher than 70%.

### Data analysis

The pharmacokinetic parameters were calculated by non–compartmental analysis using PK Solution 2.0 software (Summit Research Service). The following pharmacokinetic parameters were reported: maximal plasma concentration (C_max_), time to reach maximal plasma concentration (T_max_), area under the curve from time 0 to 24 h (AUC_0–t_), area under the curve from time 0 to infinity (AUC_0–inf_), mean resident time (MRT), volume of distribution (V_d_), total clearance (CL), and elimination half–life (T_1/2_). The absolute oral bioavailability of oxyresveratrol was calculated as (AUC_p.o._/dose_p.o._)/(AUC_i.v._/dose_i.v._). The tissue to plasma ratio (Kp) of oxyresveratrol was calculated as the oxyresveratrol concentration in the tissue divided by the oxyresveratrol concentration in the plasma at the same time point. The percentage recovery of oxyresveratrol was calculated by dividing the oxyresveratrol concentration found in urine or feces by the administrated dose. All pharmacokinetic parameters are reported as mean ± standard deviation (SD). All statistical analyses were conducted using SPSS version 16 (SPSS, Inc.). Comparison of statistical significance between oxyresveratrol alone and in combination with piperine was analyzed by a nonparametric method, with a *p*–value of less than 0.05 considered statistically significant.

## Results

All male Wistar rats that received oxyresveratrol alone or in combination with piperine showed normal physical appearance both pre–dose and post–dose (24 h). In addition, two markers of liver health, AST and ALT, also showed normal levels at pre–dose and post–dose for all test formulations. There were no statistically significant differences in these markers between rats administered oxyresveratrol alone or in combination with piperine. In relation to kidney markers, there were no significant changes between pre–dose and post–dose levels for all experimental groups, and all values were within the normal range for healthy rats (Table 1).

**Table 1:** Tolerability of oxyresveratrol alone and in combination with piperine

Parameters	Intravenous				Oral			
	Oxyresveratrol (10 mg/kg)		Oxyresveratrol + piperine (10 + 1 mg/kg)		Oxyresveratrol (100 mg/kg)		Oxyresveratrol + piperine (100 + 10 mg/kg)	
	Predose (0 h)	Postdose (24 h)	Predose (0 h)	Postdose (24 h)	Predose (0 h)	Postdose (24 h)	Predose (0 h)	Postdose (24 h)
AST (U/L)	42.00 ± 7.15	45.40 ± 3.78	34.60 ± 17.03	46.75 ± 5.57	32.40 ± 16.47	40.83 ± 22.39	17.66 ± 13.50	23.66 ± 17.32
ALT (U/L)	5.00 ± 0.00	5.80 ± 1.09	9.40 ± 1.67	7.50 ± 2.88	9.40 ± 9.28	6.83 ± 3.25	7.00 ± 2.28	11.40 ± 11.63
Creatinine (mg/dL)	0.25 ± 0.15	0.32 ± 0.08	0.22 ± 0.03	0.24 ± 0.08	0.17 ± 0.01	0.17 ± 0.01	0.22 ± 0.03	0.20 ± 0.01

Data are presented as mean ± S.D. (*n* = 6)

**Fig. 2:**
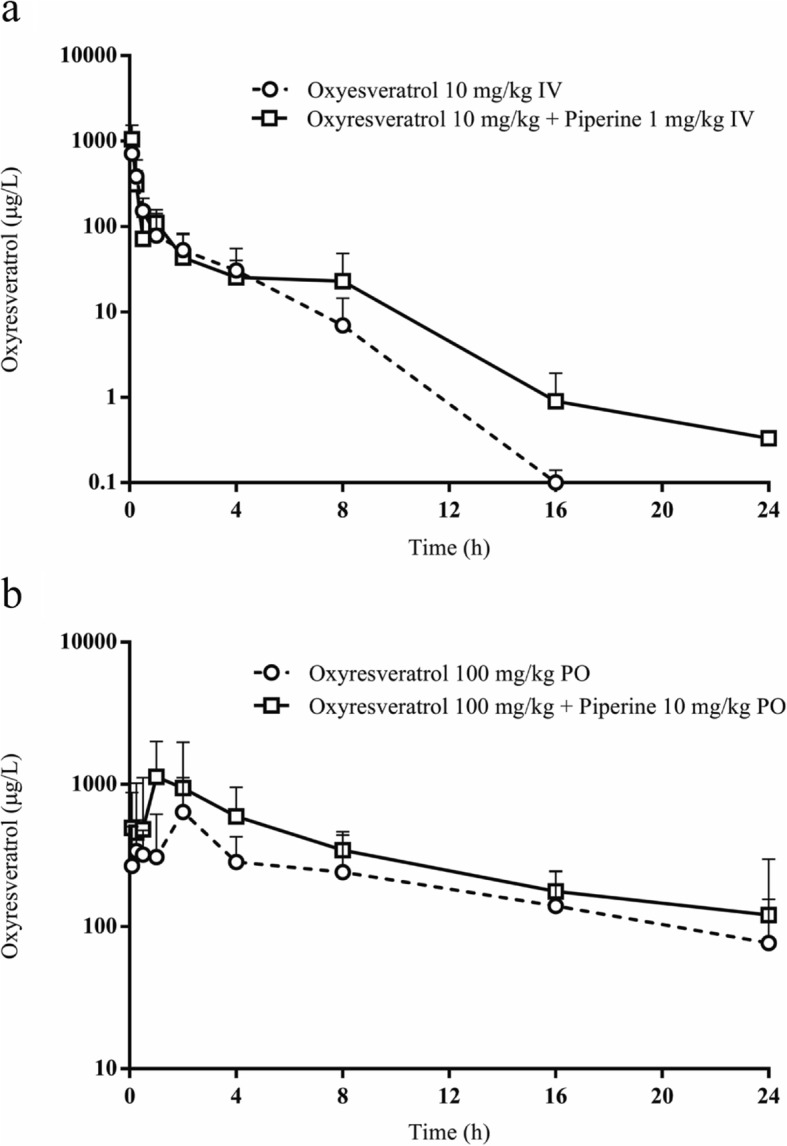
Plasma concentration–time profile of oxyresveratrol after intravenous dose (**a**); after oral dose (**b**)

The mean plasma concentration–time profiles for oxyresveratrol alone and in combination with piperine are shown in Fig. 2. The combination of oxyresveratrol and piperine resulted in higher levels of plasma oxyresveratrol, especially 8–24 h after intravenous administration. Interestingly, oral gavage of the combination led to a higher level of plasma oxyresveratrol from 5 min until 24 h after dosing. The oral combination of oxyresveratrol and piperine had a significantly higher C_max_ and tended to have shorter T_max_ values for oxyresveratrol. Similarly, all AUC values for oxyresveratrol in combination groups were higher than groups administered oxyresveratrol alone (*p* < 0.05). The mean residence time for oxyresveratrol when combined with piperine also tended to be longer, especially when administered orally (11.66 vs. 7.25 h). The combination with piperine could increase oral bioavailability of oxyresveratrol approximately 2 fold (Table 2).

**Table 2:** Pharmacokinetic parameters of oxyresveratrol alone and in combination with piperine

Parameters	Intravenous		Oral	
	Oxyresveratrol alone (10 mg/kg)	Oxyresveratrol + piperine (10 + 1 mg/kg)	Oxyresveratrol alone (100 mg/kg)	Oxyresveratrol + piperine (100 + 10 mg/kg)
Oxyresveratrol				
C_max_ (μg/L)	N/A	N/A	977.99 ± 649.59	1580.99 ± 674.31*
T_max_ (h)	N/A	N/A	2.08 ± 1.11	1.30 ± 0.67
AUC_0-t_ (μg.h/L)	825.60 ± 545.26	1455.90 ± 1953.48	5133.32 ± 1227.78	7837.18 ± 2603.81*
AUC_0-inf_ (μg.h/L)	825.80 ± 545.18	1471.00 ± 1945.62	5431.21 ± 1022.82	9375.27 ± 1974.32*
MRT (h)	1.40 ± 0.29	1.60 ± 0.42	7.25 ± 4.07	11.66 ± 8.11
V_d_ (L/kg)	47.30 ± 42.97	68.60 ± 60.02	105.10 ± 73.82	138.10 ± 112.83
CL (L/h/kg)	16.90 ± 9.30	14.60 ± 8.57	19.05 ± 4.06	11.09 ± 2.52
T_1/2_ (h)	1.70 ± 0.77	2.80 ± 1.60	3.72 ± 1.95	8.67 ± 6.55
Relative bioavailability (%)	100	178	100	173
Oxyresveratrol glucuronide				
AUC_0-t_ (μg.h/L)	20,875.50 ± 19,742.93	1190.60 ± 231.25	10,518.33 ± 6239.13	6977.34 ± 811.94
AUC_0-inf_ (μg.h/L)	20,875.80 ± 19,742.99	1640.00 ± 743.29	10,993.61 ± 6140.74	7491.24 ± 522.99
Ratio of AUCoxyresveratrol glucuronide/AUCoxyresveratrol	25.28	1.11	2.02	0.80*

Data are presented as mean ± S.D. (*n* = 6), *p < 0.05 for Oxyresveratrol alone vs Oxyresveratrol + piperine

**Fig. 3:**
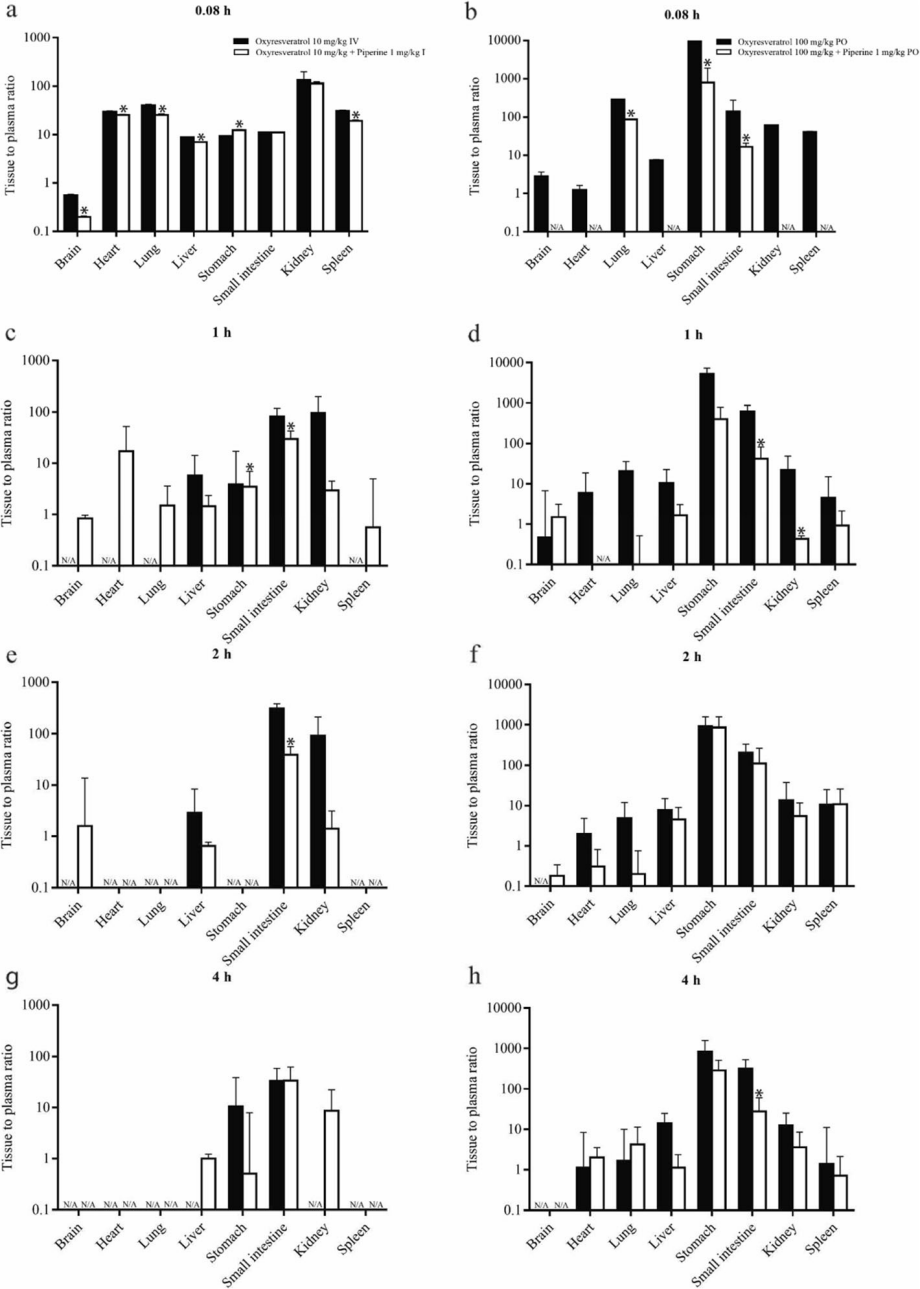
Tissue to plasma ratios of oxyresveratrol alone (black bars) and in combination with piperine (white bars) after intravenous dosing (**a**, **c**, **e**, **g**) after oral dosing (**b**, **d**, **f**, **h**)

The tissue to plasma ratio (K_p_) of oxyresveratrol alone and in combination with piperine is shown in Fig. 3. It was found that oxyresveratrol could reach most of the internal organs with a K_p_ of approximately 10–100 fold within 5 min, after which the K_p_ gradually declined at 1, 2 and 4 h after intravenous administration. For oral administration, a high K_p_ was detected in the stomach and small intestine from 5 min to 4 h. Following oral gavage, the tissue to plasma ratios of oxyresveratrol in other internal organs were approximately 1–100 fold from 5 min to 4 h. Surprisingly, the tissue to plasma ratio of oxyresveratrol in the brain was increased when administered as a combination with piperine.

**Fig. 4:**
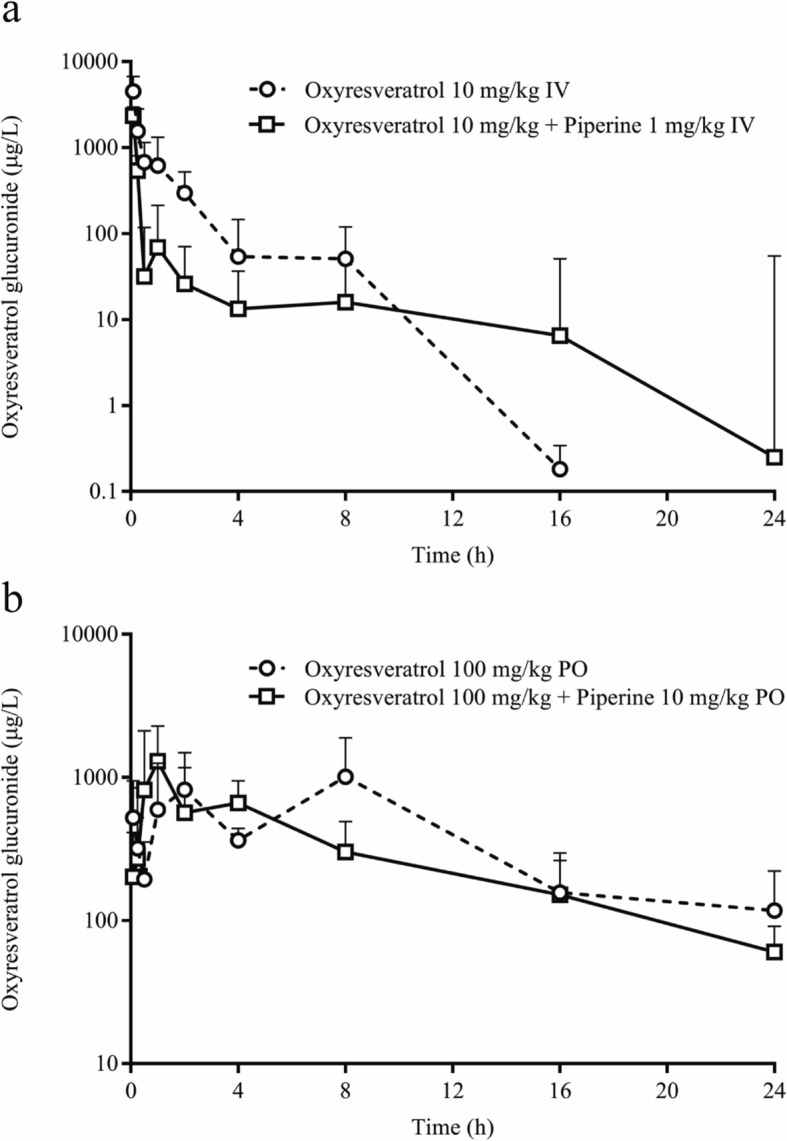
Plasma concentration–time profile of oxyresveratrol glucuronide after intravenous dose (**a**); after oral dose (**b**)

The plasma concentration–time profile of oxyresveratrol glucuronide is shown in Fig. 4. The addition of piperine appeared to reduce the production of glucuronide metabolites from oxyresveratrol after intravenous dosing. The conversion ratio of oxyresveratrol was approximately 25 following intravenous dosing of oxyresveratrol alone, which was decreased to 1.11 following dosing with oxyresveratrol combined with piperine. For oral dosing, the conversion ratio of oxyresveratrol glucuronide also decreased after oral dosing of oxyresveratrol combined with piperine (Table 2). The percentage recovery of unchanged oxyresveratrol in urine was found to be approximately 5–10% of the intravenous dose. A negligible amount (< 1%) of unchanged oxyresveratrol was found in feces from 0 to 48 h after intravenous dosing. A significant amount of oxyresveratrol glucuronide, ranging from 10 to 30%, was found in urine from 0 to 48 h after intravenous administration. A minimal amount (< 1%) of oxyresveratrol glucuronide was found in feces after both oral and intravenous dosing from 0 to 48 h (Table 3).

**Table 3:** Percent recovery of oxyresveratrol alone and in combination with piperine

Recovery (%)	Intravenous		Oral	
	Oxyresveratrol (10 mg/kg)	Oxyresveratrol + piperine (10 + 1 mg/kg)	Oxyresveratrol (100 mg/kg)	Oxyresveratrol + piperine (100 + 10 mg/kg)
Unchanged Oxyresveratrol				
Urine 0-24h	8.51 ± 7.07	5.06 ± 3.22	3.70 ± 3.41	0.88 ± 0.46
Urine 24-48h	2.87 ± 1.34	1.78 ± 1.33	0.32 ± 0.08	0.27 ± 0.18
Feces 0-24h	0.23 ± 0.20	0.17 ± 0.53	0.21 ± 0.17	0.24 ± 0.43
Feces 24-48h	0.17 ± 0.16	0.11 ± 0.01	0.11 ± 0.06	0.10 ± 0.03
Oxyresveratrol glucuronide				
Urine 0-24h	29.44 ± 28.07	10.55 ± 9.71	9.89 ± 6.90	7.81 ± 6.68
Urine 24-48h	1.39 ± 0.73	0.71 ± 0.57	3.07 ± 2.69	0.09 ± 0.05*
Feces 0-24h	0.20 ± 0.16	0.63 ± 0.58	0.19 ± 0.16	0.10 ± 0.08
Feces 24-48h	0.30 ± 0.29	0.18 ± 0.01	0.20 ± 0.18	0.14 ± 0.06

Data are presented as mean ± S.D. (*n* = 6), *p < 0.05 for Oxyresveratrol alone vs Oxyresveratrol + piperine

## Discussion

Several studies have reported that oxyresveratrol has pharmacological activity with minimal toxicity. However, this compound poses a challenge for development into a phytopharmaceutical product due to its poor pharmacokinetic profile. This problem is a common phenomenon of lead compounds from natural resources, especially low oral bioavailability due to extensive metabolism in the gastrointestinal tract [11, 14, 24]. The goal of this study was to determine whether the pharmacokinetic profile of oxyresveratrol could be improved by combination with piperine, which acts as a bioenhancer in rats. During the study, all rats showed good tolerability to oral gavage and intravenous administration of oxyresveratrol, alone and in combination with piperine. There were no significant changes in physical appearance or signs of toxicity in rats in all experiments. Two markers of liver health, AST and ALT, as well as a marker of kidney health were found to be stable and within normal ranges at pre–dose and post–dose (24 h). This tolerability result implies that all test formulae had a good safety profile in the test animals, which is consistent with previous reports by several different researchers [12, 25, 26].

As evidenced by the plasma concentration–time profiles, intravenous administration of oxyresveratrol plus piperine led to higher levels of oxyresveratrol, especially during the elimination phase from 8 to 24 h. Intravenous dose was reduced to 10% of oral dose in order to mimic systemic exposure of oxyresveratrol and piperine in rats. Therefore, calculation of pharmacokinetic parameter had more reliability from similar systemic exposure between the two routes of administration. Oral gavage of the combination showed a similar pattern, with higher levels of oxyresveratrol during the absorption, distribution and elimination phases compared to oxyresveratrol alone. This result indicates that piperine could improve the pharmacokinetic profiles of oxyresveratrol administered both intravenously and orally. Firstly, the addition of piperine led to a significant increase in oxyresveratrol C_max_ and AUC, which was approximately 1.5–fold higher, with a shorter T_max_ from 2.08 to 1.30 h. Similarly, Johnson et al. [16] reported that piperine improved the resveratrol level, evidenced by an increase in both C_max_ and AUC by up to 229 and 1544%, respectively. Resveratrol is a well–known compound with a similar structure to oxyresveratrol; however, it presents pharmacokinetic problems due to its extensive phase II metabolism [27–30]. Piperine could act as a bioenhancer through several mechanisms, such as by reducing UGT metabolism, inhibiting the efflux of P–glycoprotein, and by increasing the permeability of enterocytes [16, 20, 31–33]. In rats administered oxyresveratrol combined with piperine, a shorter T_max_ and higher C_max_ could be clearly observed during the absorption phases. Prolonged MRT of oxyresveratrol was also detected with this combination, especially after oral dosing. Interestingly, our study showed a shorter T_max_ and higher C_max_ for oxyresveratrol in both formulae compared with other pharmacokinetic studies of oxyresveratrol. This might be due to the fact that the test formulation used in our study was a clear solution, which would be more readily absorbed than the suspensions used by other studies. The combination with piperine could increase the relative bioavailability of oxyresveratrol 173%. Our finding showed a similar result to Sangsen et al. [15] that SMEDDS could improve the relative bioavailability of oxyresveratrol by 7.9–fold. Both physical modifications by SMEDDS and an addition of bioenhancers generated a superior pharmacokinetic profile of oxyresveratrol than conventional formulation alone.

In our study, we measured the oxyresveratrol level in several internal organs after intravenous and oral administration. After intravenous administration, the test compound had a V_d_ of approximately 50 L/kg, which is considered to be a large distribution volume. This might be due to the molecular weight of oxyresveratrol of 224.07 Da and XLogP 2.8, which is considered a small lipophilic molecule. Therefore, the test compound could rapidly reach the internal organs within 5 min with a K_p_ of 10–100, except for the brain. Very low levels of oxyresveratrol were observed in the brain at 1, 2, and 4 h after intravenous administration of oxyresveratrol, suggesting that oxyresveratrol has limited deposition in brain tissue. Interestingly, combination with piperine could promote oxyresveratrol level in brain tissue especially at 1–2 h after administration. Mei et al. [14] reported that oxyresveratrol is a substrate of P–glycoprotein, an efflux transporter located at the blood–brain barrier. The minimal amount of oxyresveratrol in the brain tissue might be due to restricted xenobiotic penetration and efflux transport of oxyresveratrol from the brain tissue into the circulation. The addition of piperine could improve oxyresveratrol level in the brain tissue, especially in intravenous administration.

In a previous study, oxyresveratrol was reported to be mainly metabolized by the glucuronidation reaction [14, 22]. In our study, the conversion ratio of oxyresveratrol to glucuronide metabolites was 25.28 after intravenous administration of oxyresveratrol alone. These results demonstrate that oxyresveratrol was biotransformed into glucuronide metabolites at a high ratio. When combined with piperine, this conversion ratio was decreased to 1.11 after intravenous dosing and 0.80 after oral dosing. These results indicate that piperine reduced the glucuronide reaction of oxyresveratrol in test animals. Atal et al. [19] reported that piperine at doses of 10 and 25 mg/kg p.o. in mice could inhibit UGT activity by 36 and 55%, respectively. The major route of oxyresveratrol excretion appears to be via the urinary system. Higher percentages of unchanged oxyresveratrol and oxyresveratrol glucuronide were detected in urine from 0 to 48 h after dosing. Huang et al. [22] also reported that oxyresveratrol was readily excreted in urine from 0 to 12 h after administration, based on the fact that oxyresveratrol becomes more hydrophilic by biotransformation via metabolic conjugation to monoglucuronide oxyresveratrol. Similarly, our study found a higher percentage of oxyresveratrol glucuronide in urine than unchanged oxyresveratrol.

## Conclusions

In conclusion, the addition of piperine enhanced some of the pharmacokinetic properties of oxyresveratrol via both intravenous and oral administration methods. Oral administration of oxyresveratrol combined with piperine was associated with increased C_max_, AUC and MRT. Improvement of oxyresveratrol in brain tissue was observed after intravenous administration. Glucuronide metabolites of oxyresveratrol were significantly reduced when rats were administered oxyresveratrol plus piperine. Urinary excretion of oxyresveratrol glucuronide appears to be the major route of oxyresveratrol excretion. This pharmacokinetic data will be useful for further development of oxyresveratrol as a future phytopharmaceutical product.

## Additional file

Additional file 1: **Figure S1.** LC–MS/MS chromatograms. Blank plasma spiked with 250 ng/mL oxyresveratrol (A); Blank plasma spiked with 25 ng/mL piperine (B); Plasma sample collected from rat at 1 h after intravenous administration of oxyresveratrol 10 mg/kg (C); Plasma sample collected from rat at 1 h after intravenous administration of piperine 1 mg/kg (D). **Table S1.** The precision and accuracy for oxyresveratrol determined by LC–MS/MS. **Table S2.** The recovery of oxyresveratrol after extraction from rat plasma. **Table S3.** The stability of oxyresveratrol at different storage conditions. (PDF 204 kb)

## Data Availability

The datasets used and/or analyzed during the current study are available from the corresponding author on reasonable request.

## Abbreviations

ALT: Alanine aminotransferase
AST: Aspartate aminotransferase
AUC: Area under the curve
Cl: Clearance
C_max_: maximal plasma concentration
DMSO: Dimethyl sulfoxide
i.v.: intravenous
IS: Internal standard
Kp: tissue to plasma ratio
LC–MS/MS: Liquid chromatography tandem mass spectrometry
MRT: Mean resident time
NSS: Normal saline solution
OXY: Oxyresveratrol
p.o.: per os
PIP: Piperine
SMEDDS: Self–emulsifying drug delivery system
T_1/2_: elimination half–life
T_max_: Time to reach maximal plasma concentration
UGT: Uridine glucuronosyl transferase
V_d_: Volume of distribution
XlogP: partition coefficient
