# Supplementary material for: Comparative pharmacokinetics of oxyresveratrol alone and in combination with piperine as a bioenhancer in rats
Source: BMC Complement Altern Med. 2019 Sep 2;19:235. doi: 10.1186/s12906-019-2653-y (PMC6720094; doi:10.1186/s12906-019-2653-y)
Supplement: Supplementary file 1 — Figure S1. LC–MS/MS chromatograms. Blank plasma spiked with 250 ng/mL oxyresveratrol (A); Blank plasma spiked with 25 ng/mL piperine (B); Plasma sample collected from rat at 1 h after intravenous administration of oxyresveratrol 10 mg/kg (C); Plasma sample collected from rat at 1 h after intravenous administration of piperine 1 mg/kg (D). Table S1. The precision and accuracy for oxyresveratrol determined by LC–MS/MS. Table S2. The recovery of oxyresveratrol after extraction from rat plasma. Table S3. The stability of oxyresveratrol at different storage conditions. (PDF 204 kb) [file 12906_2019_2653_MOESM1_ESM.pdf]

Additional material

Comparative pharmacokinetics of oxyresveratrol alone and in combination with piperine as a bioenhancer in rats

Dhirarin Junsaeng [1*], Tosapol Anukunwithaya [1*], Phanit Songvut [1], Boonchoo Sritularak [2], Kittisak Likhitwitayawuid [2], Phisit Khemawoot [3,4]

Affiliations

[1] Department of Pharmacology and Physiology, Faculty of Pharmaceutical Sciences, Chulalongkorn University, Bangkok, Thailand

[2] Department of Pharmacognosy and Pharmaceutical Botany, Faculty of Pharmaceutical Sciences, Chulalongkorn University, Bangkok, Thailand

[3] Department of Biochemistry and Microbiology, Faculty of Pharmaceutical Sciences, Chulalongkorn University, Bangkok, Thailand

[4] Preclinical Pharmacokinetics and Interspecies Scaling for Drug Development Research Unit, Chulalongkorn University, Bangkok, Thailand

Correspondence

Phisit Khemawoot, Ph.D.

Department of Biochemistry and Microbiology, Faculty of Pharmaceutical Sciences, Chulalongkorn University, Bangkok, Thailand 10330

E-mail: phisit.k@chula.ac.th; Phone: +66 22188378

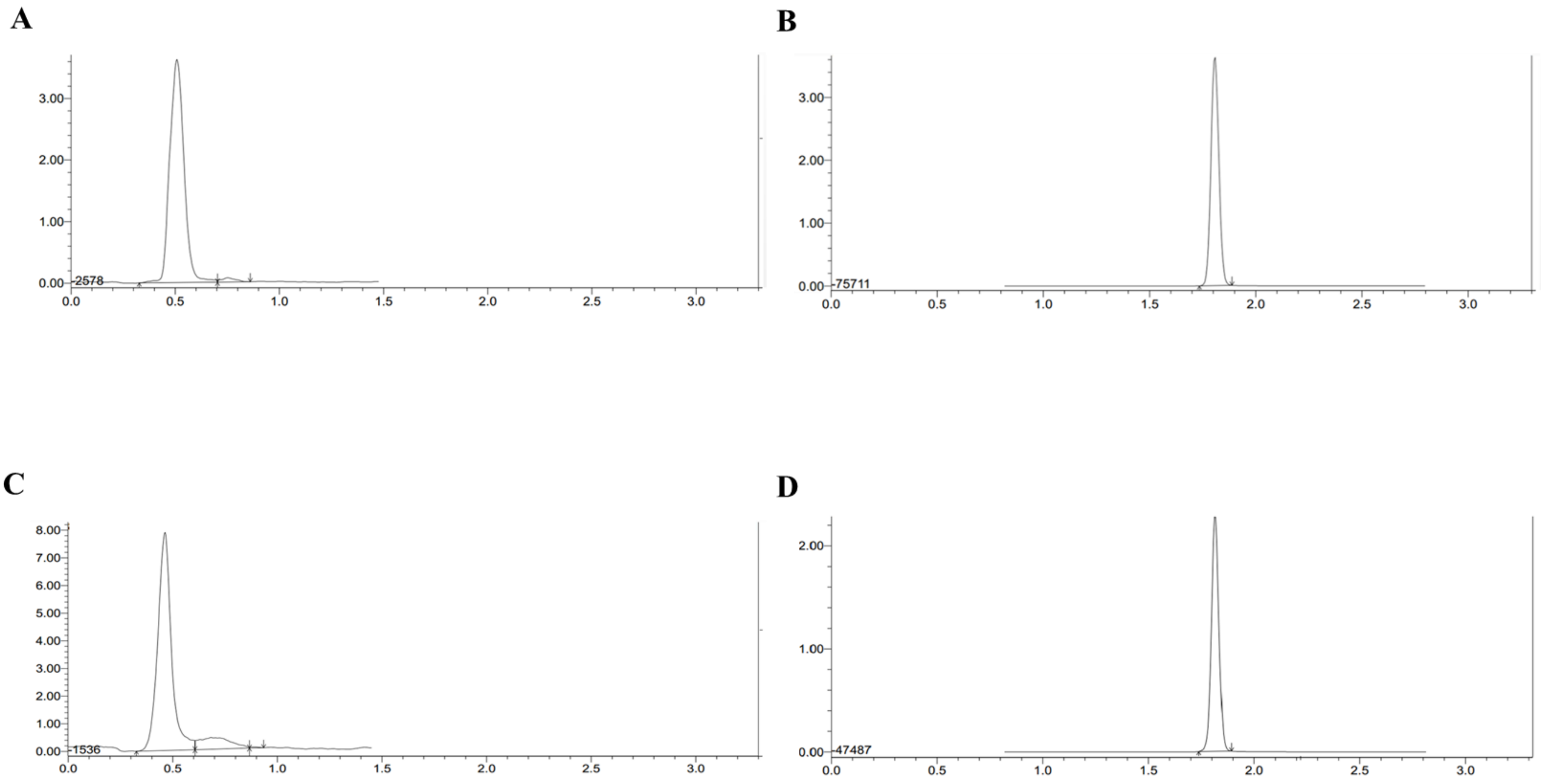

Figure 1S LC–MS/MS chromatograms. Blank plasma spiked with 250 ng/mL oxyresveratrol (A); Blank plasma spiked with 25 ng/mL piperine (B); Plasma sample collected from rat at 1 h after intravenous administration of oxyresveratrol 10 mg/kg (C); Plasma sample collected from rat at 1 h after intravenous administration of piperine 1 mg/kg (D).

Table 1S The precision and accuracy for oxyresveratrol determined by LC–MS/MS.

QC concentration (ng/mL)	Precision (%CV)		Accuracy (%RE)	
	Within–batch	Between–batch	Within–batch	Between–batch
10	7.12	9.75	–5.30	–6.94
100	2.25	3.11	–3.71	–4.63
400	1.09	2.03	–14.34	–13.39

CV, coefficient of variation; RE, relative error.

Table 2S The recovery of oxyresveratrol after extraction from rat plasma.

Concentration of OXY (ng/mL)		Mean	SD	%CV	Absolute Recovery
LQC 10 ng/mL	Un–extract	9.59	1.64	17.11	104.57
	Extract	10.02	1.15	11.49	
MQC 100 ng/mL	Un–extract	108.14	2.88	2.66	88.74
	Extract	95.96	1.60	1.67	
HQC 400 ng/mL	Un–extract	393.02	11.53	2.93	90.43
	Extract	355.42	7.60	2.14	

LQC, low concentration quality control; MQC, medium concentration quality control; HQC high concentration quality control; CV, coefficient of variation

Table 3S The stability of oxyresveratrol at different storage conditions

Conditions	QC concentration (ng/mL)	Measured Value (ng/mL)	SD	%CV	%RE	%Recovery
Room temperature (12 h)	10	6.35	0.26	4.11	36.51	63.49
	100	73.50	1.70	2.31	26.50	73.50
	400	272.61	6.39	2.34	31.85	68.15
Autosampler	10	8.77	1.09	12.44	12.31	87.69
	100	92.60	1.86	2.01	7.40	92.60
	400	345.41	5.06	1.46	13.65	86.35
Three Freez–thaw cycles	10	5.87	0.69	11.79	41.26	58.75
	100	72.84	3.62	4.98	27.16	72.84
	400	281.73	10.00	3.55	29.57	70.43
Storage at –20°C (30 d)	10	7.24	0.85	11.77	27.60	72.41
	100	85.78	3.94	4.59	14.22	85.78
	400	324.47	17.08	5.26	18.88	81.12

QC, Quality control; CV, coefficient of variation; RE, relative error.
